# The Role of Experts in the Covid-19 Pandemic and the Limits of Their Epistemic Authority in Democracy

**DOI:** 10.3389/fpubh.2020.00356

**Published:** 2020-07-14

**Authors:** Andrea Lavazza, Mirko Farina

**Affiliations:** ^1^Neuroethics, Centro Universitario Internazionale, Arezzo, Italy; ^2^Institute of Humanities and Social Sciences, Innopolis University, Innopolis, Russia; ^3^Department of History, Philosophy, and Religious Studies, Nazarbayev University, Nur-Sultan, Kazakhstan

**Keywords:** SARS-CoV-2, public health, political process, expertise, herd immunity, disabled people

## Abstract

In the 2020 Covid-19 pandemic, medical experts (virologists, epidemiologists, public health scholars, and statisticians alike) have become instrumental in suggesting policies to counteract the spread of coronavirus. Given the dangerousness and the extent of the contagion, almost no one has questioned the suggestions that these experts have advised policymakers to implement. Quite often the latter explicitly sought experts' advice and justified unpopular measures (e.g., restricting people's freedom of movement) by referring to the epistemic authority attributed to experts. The main goal of this paper is to analyze the basis of this epistemic authority and the reasons why in this case it has not been challenged, contrary to the widespread tendency to devalue expertise that has been observed in recent years. In addition, in relation to the fact that experts' recommendations are generally technical and supposedly neutral, we note that in the COVID-19 crisis different experts have suggested different public health policies. We consider the British case of herd immunity and the US case of the exclusion of disabled people from medical care. These decisions have strong axiological implications and affect people profoundly in very sensitive domains. Another goal is, therefore, to argue that in such cases experts should justify their recommendations-which effectively become obligations-by the canons of public reason within the political process because when values are involved it is no longer just a matter of finding the “best technical solution,” but also of making discretionary choices that affect citizens and that cannot be imposed solely on the basis of epistemic authority.

## Introduction: Experts and the COVID-19 Pandemic

A case of coronavirus (SARS-CoV-2) causing severe acute respiratory syndrome (SARS) was first (officially) identified in the Chinese city of Wuhan, Hubei Province, in December 2019. The virus can be transmitted between people who are in proximity to one another and via respiratory droplets produced when an infected patient coughs or sneezes. The virus is also transmitted when someone touches an object with the virus on it. The outbreak initially spread mostly within mainland China. On February 12, 2020, the World Health Organization (WHO) officially named the disease caused by the novel coronavirus as Coronavirus Disease 2019 (Covid-19). By the end of February 2020, Covid-19 had infected more than 75,000 people. During the next months, new major epidemic foci of Covid-19 were identified and started to rapidly grow in Asia (especially in India), in Europe (especially in Italy, United Kingdom, Spain, France, and Germany), in North America (especially in the US), and in the Middle East (especially in Iran and Saudi Arabia), with an increasing number of confirmed cases in Latin America (especially in Brazil). Based on these alarming levels of spread and severity, on March 11th 2020 the World Health Organization described the Covid-19 situation as a pandemic. As of July 2020, more than 10 million cases of Covid-19 were reported resulting in about 500,000 deaths^1^.

As the emergency worsened, it became clear that the leaders of many countries initially underestimated the severity of the pandemic. In the first few weeks, there was a notable lack of information characterized by the inability or unwillingness to provide precise information about the spread of the virus on the part of several governmental agencies. For instance, China hid and censored the reports released by the doctors, who first became aware of the spread of a dangerous new virus. In this case, what was at play was the political will not to frighten the population and avoid economic repercussions, especially on exports, in a city like Wuhan, home to important manufacturing companies that have strong trade relations with the whole world^2^. Secondly, those in power showed general unpreparedness to manage the crisis, once the pandemic could no longer be denied or hidden.

In addition to the will of not inducing panic or creating economic hardship, the concern of some state authorities was to show that they were in full control of the situation by not having to introduce extraordinary measures, which are a sign of a lack of preventive interventions or ineffective ordinary containment. This has caused many affected countries to fall short of delivering unambiguous advice on when and how to limit gatherings, cancel big events, postpone travel, or reduce industrial production and trade, which contributed substantially to the spread of the infection. For example, Donald Trump's dismissed the pandemic as a “Democratic ‘hoax,” predicting that it would disappear like a miracle. Likewise, the Brazilian president Jair Bolsonaro characterized the coronavirus pandemic as a media-fueled “fantasy.” More generally, many politicians have consistently engaged in a dangerous game of reality-denial that so far has cost many thousands of lives and is bound to cost more^3^.

In the meantime, though, especially in countries where the media are free and able to provide complete information in real-time, the population became aware of the danger posed by Covid-19^4^. Within this framework, medical experts (especially virologists, immunologists, and epidemiologists but also statisticians and public health scholars) have been stepping up. Unlike politicians and decision-makers at various level, who have been offering rather vague and often contradictory advice from the onset of the pandemic, many experts have been warning for weeks that the outbreak could explode and suggested very early to put in place a range of hard measures (including social distancing, closures of schools and universities, bans on large gatherings and international travels, smart working, and self-confinement) to prevent the virus from spreading further (1, 2).

Thus, for their resolution and proactiveness in the face of the growing number of deaths, experts have quickly gained general appreciation in society and acquired an increasingly central role in counteracting the spread of the disease. Politicians (prime ministers, presidents, ministers, members of parliaments), who at the onset of the pandemic often lacked leadership, started calling upon experts to help devise the best possible strategies to protect society and public health. The media also started giving experts a prominent role, hosting panels of experts in TV debates aimed at informing the public about the causes of the pandemic and the possible preventative measures to be taken in order to avoid contagion. Thus, after realizing the seriousness of the situation, the dangerousness of the disease, and the extent of the contagion, most of the people began trusting experts more than their elected representatives.

In general, though, we can say that the medical field is one of the domains where people rely on experts for decisions concerning their health and safety. Recently, the rejection of vaccines and the popularity of treatments alternative to those recommended by mainstream medicine have gained attention; however, this phenomenon of refusal of mainstream medicine remains rather limited and it is not shared in the wider society (3)^5^. Experts still maintain their epistemic authority among the public in the biomedical field and this epistemic authority is mainly based on the fact that therapies are becoming more and more effective, that some diseases have been eradicated (e.g., smallpox, rinderpest), and that average life expectancy has substantially increased over the last 50 years^6^.

In the context of the coronavirus pandemic, most world leaders began appealing to medical experts and to their epistemic authority to justify the implementation of unpopular measures (such as enforced quarantine) considered the most suitable to slow down the spread of Covid-19^7^. This step has been motivated by, at least, two elements. On the one hand, political authorities perceived that their ordinary actions were ineffective and had to make full use of biomedical expertise, often essentially delegating strategies and decisions to experts (e.g., implementing them and resolving any conflicts between different social actors; for example between trade unions and employers, the former being more favorable to closing factories for the workers' safety, the latter more inclined to keep them open for economic reasons). On the other hand, if leaders resort to the epistemic authority of experts, they are prima facie relieved of responsibility for the choices made, especially if they are unwelcome by public opinion, are ineffective, or have unforeseen negative side effects. In reality, this dynamic that leads experts to assume a central role in politics can -as we shall see below- create problems in itself, since the strategies proposed by experts are often far from neutral with respect to the values that a pluralistic society considers relevant. In this paper, we explore the ramifications of this idea, which has been overlooked in the relevant literature.

As our study is not an empirical one, we resort to qualitative analysis, which involve two cases, namely the early management of epidemic in the UK, and the limited access to life-saving therapies for disabled people in the US. We present a theoretical framework of experts' epistemic authority and introduce philosophical and normative considerations to try to determine the extent to which the authority of experts should be followed. Crucially, these considerations are based on facts and events that are publicly available but not well-scrutinized so far. Accordingly, the structure of the paper is the following (necessarily different from the classical structure and partition of a quantitative study article).

In section The State of Affairs: Epistemic Authority, Experts, and Their (Controversial) Role, we lay out the basis of our study; that is, we analyze the basis of the experts' epistemic authority and the specific dynamics at stake in the case of the coronavirus pandemic. In section Case Studies: Expert Authority and Non-neutral Assessments, we consider the role of experts' recommendations in society in the light of the two cases we discuss. We show that these recommendations should not automatically become obligations simply because of the experts' epistemic authority; rather, they ought to be discussed thoroughly, based on the canons of public reason within the political process, so as to reach the broadest possible consensus. In section Discussion: What we Can Learn From the Responses to the Pandemic, we consider what we can take home from the two cases analyzed and offer a range of suggestions about the future role of biomedical experts in pandemic situations. We conclude the paper, section Conclusion, by summarizing what we have achieved and by reflecting on the implications of our findings.

## The State of Affairs: Epistemic Authority, Experts, and Their (Controversial) Role

According to Goldman [(4), p. 92]: “[W]e can say that an expert (in the strong sense) in domain D is someone who possesses an extensive fund of knowledge (true belief) and a set of skills or methods for apt and successful deployment of this knowledge to new questions in the domain. Anyone purporting to be a (cognitive) expert in a given domain will claim to have such a fund and set of methods, and will claim to have true answers to the question(s) under dispute because he has applied his fund and his methods to the question(s).” For Goldman, someone is an expert as long as she satisfies two basic properties: (a) she knows a lot about a given topic; (b) she can apply that extensive knowledge of that given topic to other situations, so as to rationally predict their possible outcomes. Goldman's view of expertise is sound and has been very influential; however, it is not the only one available in the literature.

Another account of expertise, one that is perhaps more relevant for the purpose of this paper, was recently developed by Quast (5), who argued that the nature and value of expertise lie in its service-function and social role. According to Quast, expertise is a social kind that not only requires competences, relevant knowledge and the capacity to apply this knowledge and competences to new situations (as in Goldman's account) but also inevitably requires and demands a special responsibility toward society – a deontic dimension, so to speak. Thus, experts – according to Quast – are people who have knowledge and competences, which they can apply to new scenarios, but who also have a specific mission in society. By virtue of this, experts possess an improved epistemic stance (or greater epistemic authority) over non-experts and can make informed decisions and accurate predictions that can increase the welfare of their communities. Here welfare is understood in the broadest sense of the term, including both knowledge and the material and social living conditions of people (e.g., through improved functioning of political institutions or more inclusive policies).

Yet, what are the specific traits that allow experts to acquire such an improved epistemic stance? In other words, what are the markers of expertise and when can we reasonably say that we can trust someone as an expert? These questions are hard to answer and probably there isn't a clear-cut solution to them. However, we can say that someone can be considered as an expert if she has, at least, a combination of the following traits: (i) motivation and focus; (ii) good education; (iii) solid experience in the field; (iv) significant achievements; (v) excellent reputation among peers; (vi) a prestigious position; and (vii) no personal interest in the issue at stake.

In other words, an expert is a person that typically has a high academic degree (such as a Master's or PhD) from a reputable institution and that has significant experience in their own field. An expert, however, is also a person that has achieved significant results in her field (e.g., publications in leading journals, prizes, fellowships, or grants), that is held in high esteem among her colleagues, and that holds or has held prestigious positions (in important institutions, for instance). But an expert, in order to be trusted, also ought to be a disinterested party, meaning that she must not have any stake in a specific belief. In addition, she must be motivated and focused (6) in her research and not be willing to compromise it for immediate rewards. These are the traits that, jointly taken, can make someone an expert and can warrant them greater epistemic authority (hence trustworthiness) over non-experts. But why do people trust experts?

In general, it can be said that there are three orders of reasons why experts have gained more importance in our lives and in the public arena. The first reason is that resorting to experts actually works: it's a rather successful practice. Average life expectancy got longer thanks to medicine and its steady progress. Life conditions have progressively improved, as well. Many life problems have found a concrete solution, with more goods at people's disposal, more free time, and the possibility to travel.

The second reason is that resorting to experts is a way to curb any potential controversy. Many descriptions of science portray it as a selection/competition between theories aiming at the “true” description of reality or as a process of repeated conjectures and confutations (7); however, the most adequate image is probably that of the inference to the best explanation (8). The inference to the best explanation is a socially non-traumatic procedure aimed at progressively excluding the theories that predict and explain fewer phenomena than other ones: this leads to provisionally consider a given theory as the one with the best explicative and predictive capacity (9). Still, such theory can be refined, modified or even replaced by a more informative theory, if one is found (10).

The third reason for the experts' crucial role in democratic governments is rather cultural/intellectual. Resorting to experts and their (supposedly) objective knowledge means resorting to rationality – a faculty that, in turn, is elevated to the status of ideal objective for any evolved community, and as such is able to solve problems and controversies objectively.

Yet, being an expert and thus possessing an increased epistemic authority does not automatically warrant credibility in the eyes of laypeople. In recent years we observed a tendency to openly distrust experts and their knowledge (notable case studies involve climate change and Brexit). With respect to the latter, many foreign leaders and moral authorities had repeatedly expressed serious concerns about the possible undesirable consequences of a Brexit. In a similar vein, the UK academic community (including leading economists) consistently and overwhelmingly warned the population of the significant economic costs that leaving the EU would entail for Britain.

Such warnings were largely dismissed, and the UK left the EU on January 31st, 2020^8^. As former secretary of state for justice, Michael Gove put it: “people in this country have had enough of experts” (11). And this pattern is not only confined to the UK. In the US, voters explicitly disregarded the opinion of pundits and in 2016 elected Donald Trump, who, against perhaps 99% of scientific consensus, denies the reality of climate change (12). In France, Marine Le Pen – the leader of the National Front – routinely receives little sympathy from experts but maintains strong popular support. The same can be said for Viktor Orban in Hungary, for Geert Wilders in the Netherlands, or for the far-right coalition that is ruling Poland at the time of writing (13). Thus, it seems safe to say that everywhere, in recent years, there has been a widespread tendency among laypeople to devalue expertise, so that a very great number of people have become extremely hostile to experts.

Although this is not the focus of our article, it is important to underline that there are at least two reasons for this tendency to distrust experts, which nevertheless coexists with the massive use of experts and their knowledge in many private and public sectors^9^. The first reason, linked to the political process, is that some parties and some leaders support programmes and reforms that are based in varying proportions on nationalistic, populist, conservative and religiously inspired ideas (14). All these orientations tend to reject globalization, open economy and society, materialism, secularism, hierarchies of knowledge that exclude citizens from decision-making, progress as a primary objective, the importance of questioning and verifying one's deep-rooted convictions – in short, everything that is or appears to be the legacy of the scientific method and of the direct or indirect action of experts^10^. Another factor is the systematic exploitation of emotions and visceral responses (15) as a tool to achieve consensus on the part of politicians, which causes a further clash with the method of rational and experimental testing.

The second reason for the criticism of experts, as manifested for example in the anti-vaccination movement, has a different character, as it also affects educated and informed shares of the population in Western democracies (16). This is a fairly recent phenomenon that seems to find its main explanation in the spread of social media, i.e., in the disintermediation of knowledge and the erosion of the authority principle (17). The idea of personal autonomy can also take the form of the rejection of expert opinion as a claim to one's own space of self-determination, even if this implies one's ignorance with regard to the subject matter. In this way, we are witnessing, in some areas of knowledge and in some social contexts, a contestation of the idea of competence and the division between experts and laypeople.

Everyone, in short, has the right and the possibility to document themselves and get their own idea, thanks to easily accessible tools such as Google, and then to spread and defend their view via social media, which are a completely new and very powerful means of knowledge creation from an epistemic and social point of view. There is a claim for equality which, having spread in many other areas, is also supposed to apply in the field of knowledge. The feeling of having been deprived of decision-making power by a small group of competent people with consolidated and apparently inaccessible knowledge – essentially a sense of impotence – often provokes a hostile reaction with respect to the experts' indications, except when they are perceived as a standard and non-controversial procedure (take an analgesic against headaches; buy a fast and powerful computer, etc.) (18).

These two strands of hostility toward experts, while being differently motivated, are united by the fact that rational arguments and well-documented evidence tend not to convince those who support unorthodox positions or contest the scientific mainstream (19). This type of reaction has been attributed to evolutionary psychological mechanisms (which favor the unconscious selection of evidence in favor of one's own beliefs) and to group cohesion, which promotes the maintenance of shared views to strengthen the identity and cooperation of members, while also fostering further exposure to messages in support of the accepted ideas in the so-called “echo chambers” (20).

However, there is evidence that when the subject on which laypeople and experts disagree directly affects people's lives, with varying degrees of threat to which an effective response must be given, then the persuasive force of established expertise and knowledge prevails and is used to a much greater extent. The coronavirus has a short-term direct effect, whereas – for example – climate change affects people's lives but not in the immediate term, so the evidence is dismissed. This is particularly important in the context of the Covid-19 pandemic (21). Now, a critical ingredient for successfully addressing pandemics worldwide is public order and civil obedience to protocols. This means that, for people to respect socially demanding measures (such as enforced quarantine), they need to be offered reliable and credible messages from trusted sources of information. So, intentional disinformation about science is particularly damaging to the credibility of experts seeking to formulate appropriate health policies (consider for instance the anti-vaccination movement) as it inhibits people's trust in experts' advice.

In the COVID-19 pandemic, however, we observed -as discussed above- two stages^11^. The first stage was characterized by concealment of information and institutional disinformation, which contributed to eroding the general public's trust in governments and international institutions (this was largely in line with the tendency observed in recent years)^11^. In the second stage, instead, experts voiced concern for emergency preparedness, protested against budget cuts to essential domestic and global health programs, and begun proposing the implementation of public health measures to help citizens avoid contagion, thereby becoming – once again – sources of accurate information and of reliable health policies (22)^12^.

Due to the seriousness of the pandemic and the concrete threat to the population, with the exponential increase in the number of infections and victims observed, most of the population relied on the authority of experts^13^. In a similar vein, nearly all governments – some out of conviction and therefore more quickly, others out of necessity shortly afterwards – made use of technical-scientific committees already active or set up for the occasion, and delegated to them the identification of the most suitable public health policies. In some cases, as we shall see, expert opinions have been divergent or governments have chosen to rely on experts who were more in tune with their general approach, agenda, or public health policy. Overall, this delegation of power and of responsibility to experts has allowed leaders and governments to lighten their own responsibility toward society.

Having discussed the basis of epistemic authority, we next look at the role that experts' recommendations can play in society. We argue that such recommendations -albeit reputable and authoritative- shouldn't be accepted uncritically; rather they always ought to be discussed thoroughly in the political process, in the light of the canons of public reason.

## Case Studies: Expert Authority and Non-neutral Assessments

The epistemic authority of virologists and epidemiologists cannot only be based on the success of biomedical science, as the latter is not an exact discipline. Even if the so-called evidence-based medicine (23) has gained ground, and algorithms are proving to be better than humans in certain types of diagnoses (24), it is still said that medicine is an art, where personal experience and intuition play a key role, as shown by Dr House in the popular TV series. This requires the epistemic authority of experts to have a more solid basis. This basis seems to be scientific naturalism (25, 26) understood as a conception of reality and knowledge whose core consists of two crucial ideas or tenets:

- at the ontological level, supernatural elements do not exist,
- at the epistemological level, science (or otherwise empirical, intersubjectively reproducible and falsifiable research) is the primary, if not the only source of reliable knowledge.

To consider a famous definition, “[scientific] naturalism is a species of philosophical monism according to which whatever exists or happens is natural in the sense of being susceptible of explanation thorough methods (.) paradigmatically exemplified by the natural sciences” [(27), p. 448]. And these methods and explanations are or should be, strictly empirical. As a consequence, scientific naturalism also implies that “scientific inquiry is, in principle, our only genuine source of knowing or understanding. All other alleged forms of knowledge (e.g., a priori knowledge) or understanding are either illegitimate or are reducible in principle to scientific knowing or understanding” [(28), p. 4].

Now, on the one hand, there seems to be a growing appeal to the epistemic authority of experts (in line with the reasons presented above); on the other hand, however, in the public process scientific naturalism often ends up clashing with religious and various other moral and cultural values. In fact, the phenomena that are still to be explained in a scientific, shared and non-controversial way include central aspects of the human world, which are defined by their inherently normative and axiological nature. So, normativity potentially stands as one of the main obstacles to scientific naturalism and its claims of naturalization, as many of our decisions are based on criteria other than purely scientific ones, while being neither irrational nor unreasonable. Normativity constrains our thinking and our actions, in the sense that it presupposes that there are things we should think or do as well as assessments we should give (even if, often, we think or do something else). This fact has great importance for politics, where it takes the form of regulatory decisions made according to majority-based procedures.

In other words, science isn't in the business of answering moral questions: rather, its findings can be used to inform answers to moral questions (Lavazza and Farina, under review). But since moral questions are irreducibly normative, and since science (according to naturalists) is irreducibly non-normative, there is no chance that science can discover all truths (provided there are any normative truths). In this sense the choices suggested or directly made by experts should be mostly neutral. And if they are not, they should be justified not only by the epistemic authority of their holders but also by acceptable public reasons expressed in the political process. Ideally, in the public arena different comprehensive visions are compared and everyone can understand and accept the proposed arguments without one's (epistemic) authority being an element of relevance in the discussion. The latter, however, must remain within the canons of procedural rationality – something that is not always easy to define but which we can all intuitively understand (29, 30).

However, in some cases experts' assessments are not neutral in the sense explained above. Two examples that occurred during the Covid-19 pandemic in Britain and in the United States may be used as good illustrations of this point.

### The British Case of Herd Immunity

At the beginning of the COVID-19 pandemic, Prime Minister Boris Johnson was very skeptical about the possibility of an epidemic taking place on a large scale in Britain. For this reason, Johnson opposed the implementation of draconian measures of prevention, such as the suspension of activities already implemented or the banning of large meetings or international travels, both for economic reasons and for idealistic reasons, mostly political considerations (involving -for instance- respect for citizens' rights). However, some experts also supported Johnson's position with scientific motivations, including the Chief Scientific Adviser to the Government, Patrick John Thompson Vallance, and the UK government's chief medical adviser, Chris Whitty^14^. Whitty and Vallance initially endorsed the government's prudent strategy to fight coronavirus, based on the Contain-Delay-Mitigate-Research (31): as a result, those who had symptoms were not tested, contrary to WHO's suggestion, and the government enforced at a societal level neither quarantine nor isolation. Vallance explained that Britain needed to acquire “herd immunity;” that is, at least 60% of Britons needed to contract Covid-19 in order to develop effective antibodies and no longer transmit the disease since SARS-CoV-2 occurs seasonally.

This health strategy is based on the utilization of an established scientific fact, “herd immunity,” which is achieved when a certain proportion of the population develops antibodies to a certain infectious disease, either in order to stop the infection or keep it below a minimum threshold (32). Usually, herd immunity is achieved with the spread of a specific vaccine, as happened for example in the case of measles (33)^15^. Unlike the restrictive measures adopted in other European countries, the experts advising Johnson's government argued that such a strategy ought to be implemented to protect the elderly and the more fragile in the long term. This health policy, however, was immediately criticized by part of the scientific community and by the public as well. For some, Vallance's theory represented a huge risk that could have caused the deaths of hundreds of thousands of British people^16^. There are 67 million Britons, so 60% means about 40 million. With a lethality rate of 1% (this is a very conservative estimate), the approach suggested by Johnson could have easily resulted in about 400,000 deaths. And the British health care system could have been put under extreme pressure, with a very high number of patients admitted to intensive care for acute respiratory problems.

A working paper by the Imperial College Covid-19 Response Team (34) published on March 16, 2020, predicted Covid-19 deaths in the U.K. based on a range of policies and a range of reproduction numbers. In their worst-case scenario, which assumed a reproduction number of 2.6 and the (unlikely) absence of any control measures or spontaneous changes in individual behavior, researchers estimated 550,000 deaths. The day after the release of the report the government changed its strategy by announcing more drastic measures to prevent the contagion from spreading: school closures throughout the country and the restriction of many other activities up to the general lockdown, with the justification that scientific data had changed.

Richard Horton, the editor-in-chief of the medical journal The Lancet, commented that the attitude of the government and its medical and scientific advisors was incomprehensible (31), as was the decision to change strategy only after the Imperial college paper was released. In his view, the scientific data were the same since January and nothing had changed: in his opinion, what had happened in China and what was happening in Italy was clear enough. A journalistic inquiry conducted by the Reuters found that “the scientific committees that advised Johnson didn't study, until mid-March, the option of the kind of stringent lockdown adopted early on in China […]^17^. Britons, many of them assumed, simply wouldn't accept such restrictions.” According to the investigation, “as they watched China impose its lockdown, the British scientists assumed that such drastic actions would never be acceptable in a democracy like the UK. Among those modeling the outbreak, such stringent countermeasures were not, at first, examined^18^.” In the light of this reconstruction, the Imperial College's report did not contain figures other than those which should have been already assessed and understood by government's experts but simply made them public without political mediation^19^.

At the time of writing, facts are too recent to have sufficient sources and evidence to reconstruct the causes of the decisions by the British Government. Our discussion is only intended to highlight how the intervention of experts in health policymaking can have a huge impact that goes beyond the simple application of knowledge and expertise to the given situation in order to make predictions or suggest the best means of achieving certain ends. In the same paper mentioned above, Ferguson et al. (34) acknowledged that “the social and economic effects of the measures which are needed to achieve this policy goal [of suppressing the epidemic] will be profound.” But researchers expressly did not “consider the ethical or economic implications” of choosing an aggressive “suppression” strategy rather than milder measures aimed at “mitigation.”

As former Uk minister Rory Stewart rightly pointed out before the country took more restrictive measures: “Britain is trying to follow a theory of herd immunity. In other words, they believe it's impossible to get on top of this disease, and therefore you have to ultimately let it run through the population. That is a very, very big choice. It's not a scientific choice, it's fundamentally a political choice.” Stewart added that he thought the government had made the wrong judgement by not being transparent and said that “when the public understands that implicit in this argument is that they would rather that people died earlier to prevent more people dying later, the public will be very troubled.”

Our goal in this article is not to assess the scientific soundness of the herd immunity hypothesis. On the one hand, “there is very little evidence to support the hypothesis that herd immunity would work in this case – we are dealing with a very new virus and most evidence on herd immunity comes from the context of vaccination^20^. [And], even if there were a chance that herd immunity would work as a strategy, the timing of it would have to be perfect for it to work, which seems extremely unlikely given the lack of evidence” (35). On the other hand, it cannot yet be ruled out at the time of writing that the virus might have already infected a significant proportion of the British population, as claimed by a study conducted by a group of researchers led by Sunetra Gupta (36). According to preliminary data, <1 in a thousand of those infected with SARS-CoV-2 will develop symptoms requiring hospitalization. Most individuals develop very mild symptoms or no symptoms at all. Moreover, it is well-documented how difficult it is to make reliable and realistic predictions about the development of an epidemic due to an unknown pathogen and to implement the most effective containment strategies [see (37)].

What we want to emphasize here is that public health policies can have more or less solid scientific foundations but still have consequences that are not included within the scope of purely medical decisions. In other words, the epistemic authority of experts -in our view- is not enough to justify the implementation of a rather political decision, such as that of herd immunity when it includes all its societal consequences. In this sense, we agree with the arguments presented by Ienca and Shaw (35): “Aiming for herd immunity involves a conscious policy decision to let perhaps half a million people die – mainly people over age 70 who are much more likely to require intensive care beds and to die of the virus (the same group discriminated against in Italian guidelines on rationing intensive care provision). [And] if this were a clustered clinical trial, no ethics committee on the planet would approve a design with such weak evidence and such high risks.”

But the key point is that these decisions must have a justification that is not only epistemic, based on the knowledge that is methodologically (scientific naturalism) and empirically (observation and experiments) grounded. If the aim is to combat an epidemic with a strategy that voluntarily exposes a large number of people to contagion, then this health policy incorporates values [such as those of a utilitarian approach (38) that privileges the maximization of the overall good, even at the price of the suffering of some] that simply cannot be presumed to be imposed on a modern pluralist society. In these cases, just like political parties, religious groups or opinion movements, experts must be able to articulate their proposals in terms of reasons that are accessible to all, so that every citizen has the opportunity to evaluate and adhere to them or to reject them in the usual deliberative process carried out in the public arena in accord to the shared procedural political values (29).

For example, the well-being of the majority cannot be preferred over the absolute value of every human life, based on the extrinsic authority of the person proposing one position or another. In this sense, experts with epistemic authority are not *per-se* more entitled than others to defend a certain value or a moral principle, contrary to what happens when a technical solution has to be chosen.

This means that not even approaches opposed to the British one, such as the extremely restrictive health policy adopted by countries like Italy, China, or Kazakhstan, are in principle immune from the abovesaid considerations. Excessive caution in countering a potential threat can, in fact, exploit the epistemic authority of experts to introduce measures that violate civil liberties and rights or severely restrict the ability to exercise private business. Also, in this case, the justification for similar measures should not only be the purely technical type typically provided by medical experts. In fact, such decisions can be countered by changing empirical data, and therefore value considerations must also be taken into account and framed in the political landscape according to the canons of public reason.

### The US Case of the Exclusion of Disabled People From Care

When the Covid-19 crisis in Italy worsened (beginning of March 2020), the Italian Society of Anesthesia, Analgesia, Resuscitation and Intensive Care (SIAARTI) predicted an increase in cases of acute respiratory insufficiency (requiring hospitalization in the Intensive Care Unit) of such magnitude as to cause a strong imbalance between the population's clinical needs and the effective availability of intensive resources. Faced with this scenario, it was believed that it might be necessary to adopt “criteria for access to intensive care,” “not only in strictly clinical appropriateness and proportionality of care but also in distributive justice and appropriate allocation of limited healthcare resources^21^.”

In a scenario akin to “disaster medicine,” for which there are many concrete indications for doctors and nurses involved in difficult choices, SIAARTI proposed some “clinical ethics recommendations for the allocation of intensive care treatments, in exceptional, resource-limited circumstances.” These included “an extension of the principle of proportionality of care, allocation in a context of a serious shortage of healthcare resources,” and the “aim at guaranteeing intensive treatments to patients with greater chances of therapeutic success.” Therefore, it was a matter of favoring the “greatest life expectancy.” The need for intensive care must be integrated with other elements of “clinical suitability,” thus including the type and severity of the disease, the presence of comorbidities, the impairment of other organs and systems, and their reversibility. This means not necessarily having to follow a criterion for access to intensive care like “first come, first served.” It is implicit – underlines the document – that the application of rationing criteria is justifiable only after all the actors involved have made all possible efforts to increase the availability of resources and after every possibility of transferring patients to centers with greater availability of resources has been evaluated^22^.

This type of guidelines, where choices are left to experts in the field, may generate an understandable debate, but they fall within the competence of medical managers and do not give rise to specific disagreements because, in the face of the objective temporary impossibility of treating all patients in the best possible way, certain criteria simply must be followed. And the criteria proposed by SIAATRI, like similar criteria proposed in other countries, are recognized as reasonable and supported by the specialist knowledge of experts, who are the most qualified to make these choices, although there is always room for dissent and difference of opinion.

A different case is what happened in some US states, where some criteria have been either reconsidered or set from scratch in the face of the Covid-19 emergency. For example, at the end of March 2020, people with spinal muscular atrophy were excluded from intensive care in Tennessee. In Minnesota, cirrhosis of the liver, lung disease and heart failure were considered as diseases that had priority over Covid-19. In Michigan, precedence was given to workers employed in essential services. And in Washington State, New York State, Alabama, Tennessee, Utah, Minnesota, Colorado, and Oregon, doctors were required to assess the general physical and intellectual ability of patients with Covid-19 before intervening with resuscitation procedures.

Different approaches emerged in the strategies prepared or revised by local experts, with a common trend. Of the 36 or so states that made their criteria known, a dozen also listed considerations with respect to the intellectual capacity of patients, and others indicated precise conditions that could lead to a lesser recognition of disabled people's rights to care as opposed to other patients^23^. In the Alabama guidelines, for instance, it is claimed that “persons with severe intellectual disability, advanced dementia or severe traumatic brain injury may be poor candidates for ventilator support;” and that “persons with severe or profound intellectual disability, moderate to severe dementia, or catastrophic neurological complications such as persistent vegetative state are unlikely candidates for ventilator support^24^.”

These rules and the reference to “cognitive abilities” in the guidelines of Washington state or to “severe neurological disorders” in those of Maryland and Pennsylvania have aroused the protests of the associations for the defense of disabled people. Disability Rights Washington, Self-Advocates in Leadership and The Arc of the United States have sued the State of Washington to prevent the enactment of the criteria for access to life-saving care for Covid-19^25^. And other organizations have appealed to the federal government to impose on local authorities and hospitals the principle that disabled people are entitled to the same treatment as all other Covid-19 patients^25^.

Disabled people's associations have addressed the leaders of the Senate, and some MPs have written to the Department of Health and Justice inviting them to provide clear guidance to protect people with disabilities^26^. In the US, in fact, civil rights laws prohibit discrimination on the basis of race, color, national origin, disability, age, and sex. Subsequently, Alabama had to revoke its plan to deny ventilators to patients with cognitive disabilities in the event of a shortage of them^27^. In fact, the HHS Office for Civil Rights has determined that the plan violated federal civil rights laws.

The guidelines of individual states may reflect the positions of health experts alone or also the political orientations of legislators^28^. However, the examples given so far point to situations where proposals or decisions made by experts – based on their technical expertise and presumably in good faith, i.e., without explicit cultural, ideological, political or religious views or biases being at play – cannot be justified simply by their epistemic authority (i.e., based on the fact that experts know more than laypeople, are more effective in a particular circumstance and ground their views on scientific naturalism, which is the most reliable epistemic theory). The possible use of the disability criterion to put people with disabilities at the bottom of the list of those who can access intensive care, as shown by the reactions provoked in the United States, must be publicly justified with reference to reasons that can convince the bearers of general values and interests within society.

It is certainly necessary to decide who should be assigned a ventilator in the ICU when there are more patients than devices available. And it does not seem sensible to choose by fate or according to the extemporary judgment of the clinicians. Now, reasonable general criteria such as those exposed in the document of the Italian Society of Resuscitators can be shared and accepted on the basis of the epistemic authority of the experts. Criteria that are more controversial or that may conflict with widespread beliefs and rules (e.g., equal rights and opportunities for people with disabilities) ought instead to be proposed and argued for on the basis of reasons that do not only refer to established biomedical knowledge but also meet the requirements of procedural political discussions bound to assumptions that all citizens might reasonably share.

Having discussed these two case studies, we are now able to draw an intermediate conclusion. It appears that the role of experts is crucial in fighting an unknown pandemic as political choices can be extremely slow and ineffective. Scientists' suggestions may be unpopular and go against shared beliefs and contingent interests but are in most cases based on specific expertise that other citizens do not have. Involving experts, even preventively, can be the best strategy for legislators and decision-makers who want to defend their society from the threat of an unknown virus. In this sense, cultural and social trends aimed at devaluing the authority and role of experts in society must be countered. However, it is advisable that experts' recommendations are always discussed through the prism of public reason. We analyse this point at length in the next section of this paper.

## Discussion: What we can Learn From the Responses to the Pandemic

As we have seen above, the action of experts and scientists is not always as technical and neutral as it is supposed to be (39, 40). Experts' recommendations have sometimes strong axiological implications, involving very different treatment decisions and different sets of cultural, moral, or religious values. In such cases, experts should justify their recommendations (which effectively become obligations) by the canons of public reason within the political process. In fact, when values come into play it is no longer just a matter of finding the “best technical solution,” but also of making discretionary choices that affect citizens and that cannot be imposed solely on the basis of epistemic authority.

An example of technical recommendations that end up having a major effect on the balance of principles and rights within a liberal democracy is the tracking of people infected by Covid-19 and of those who have come in contact with them. Indeed, an effective measure to curb the epidemic seems to be to follow (and reconstruct) the real-time movements of all those who are positive and those who have been in contact with them. It is thus possible to quickly circumscribe an outbreak and prevent it from spreading because even coronavirus-negative people would know immediately which people and areas to avoid. This makes it possible both to intervene clinically in a targeted and more effective way and to act in an epidemiologically efficient way, avoiding the damaging effects of lockdown on citizens and economic activities^29^.

This method, thanks to today's technological knowledge, infrastructure and dissemination of individual devices, seems quite simple to implement and indeed it is being implemented (in countries like South Korea, for instance). It is enough to activate the GPS of each smartphone and thanks to a specific app, with the help of telephone operators, follow all the movements of the subjects “of interest” – for example, as mentioned, the person who tested positive and all those who are close to them, in order to isolate, as far as possible, the vectors of contagion^30^. Alerts to all those who are in the outer circle around the area subject to preventive “closure” make it -in principle- possible to stop the chain of virus transmission. Tests are not only carried out on symptomatic patients but also on a sample basis according to a specially designed statistical programme.

Now, let's suppose that this system is scientifically grounded and proves to be truly better than lockdown in terms of costs and benefits because it reduces overcrowding in intensive care units and is less expensive in terms of effects on the GDP. It could also be more advantageous in terms of individual rights, given that general confinement in one's own home drastically restricts the fundamental rights of movement and assembly (like a prison sentence, in many respects). However, on the one hand, digital tracking “only” nullifies the right to privacy (provided that the system is mandatory, but optional compliance also poses problems of social pressure and possible discrimination against dissidents). On the other hand, though, the risk of using the monitoring strategy is that something that is acceptable in principle may then become an unacceptable constant danger for all citizens.

In fact, when the lockdown ends, everyone can go back to their own activities and the negative effects of various kinds can be dissipated within a relatively short period of time. Once the tracking system is implemented, however, not only is the data acquired during the epidemic stored forever, but the entire tracking system becomes available for new uses. Also, it should be noted that the psychological or moral resistance to the implementation of full-scale tracking that the majority of people may have before the implementation of such measure during the pandemic may well be weakened after its actual implementation when the pandemic it's over (this is because people get used to it and may slowly forget about it). This opens to the possibility of a large swathe of people being tracked and almost automatically accepting more restrictions on their rights, which is quite problematic. So, the vulnus inflicted on the right to privacy can subsequently be transformed into a powerful mean to control citizens and to give authorities immense, unchecked, and unbalanced power.

In this sense, following Ienca and Vayena (41), we suggest that experts should propose recommendations that are: “(i) proportional to the seriousness of the public-health threat, (ii) limited to what is necessary to achieve a specific public-health objective, and (iii) scientifically justified.” In the case of personal tracking in order to reduce SARS-CoV-2 infections, an efficient technical solution may imply, as an unintended but foreseeable effect, a temporary or even prolonged shift in the political balance. This type of expert recommendation, while technically flawless, is not neutral for individuals and for society and should, therefore, be proposed and evaluated according to procedures that do not merely establish the epistemic authority of the advocates and the recommendation's adherence to scientific criteria. The values at stake are different and conflicting – the right to health, the right to privacy, political freedom – and the prevalence of one or the other should be entrusted to an assessment typical of decisions made in the political process with the participation of all citizens, usually in the forms of representative democracy. And just as we should never give up the contribution of experts, so the state of emergency and the limited time available to make an effective decision should never prevent such an assessment when axiological aspects that go beyond epistemic authority are at stake.

## Conclusion

In the 2020 Covid-19 pandemic, medical experts (virologists, epidemiologists, public health scholars and statisticians alike) have become instrumental in suggesting policies to counteract the spread of coronavirus. Given the dangerousness and the extent of the contagion, almost no one has questioned the suggestions that these experts have advised policymakers to implement. Quite often the latter explicitly sought experts' advice and justified unpopular measures (e.g., restricting people's freedom of movement) by referring to the epistemic authority attributed to experts.

In this paper, we analyzed the basis of this epistemic authority and the reasons why in this case it has not been challenged, contrary to the widespread tendency to devalue expertise that has been observed in recent years. In addition, in relation to the fact that experts' recommendations are generally technical and supposedly neutral, we noted that in the COVID-19 crisis different experts have suggested different public health policies. We considered the British case of herd immunity and the US case of the exclusion of disabled people from medical care. In those cases, decisions had strong axiological implications, deeply affecting people in very sensitive domains.

Based on our theoretical and empirical analysis, we argued that experts should justify their recommendations - which effectively become obligations-by the canons of public reason within the political process because when values are involved it is no longer just a matter of finding the “best technical solution,” but also of making discretionary choices that affect citizens and that cannot be imposed solely on the basis of epistemic authority. Epistemic authority may justify recommendations in strictly technical matters, but some decisions which are not only technical but also normative must have a shared political, cultural, and perhaps even ethical justification. We scrutinized the political and moral aspects involved the political process, in which every citizen exercising their reasonableness within the framework of liberal procedures has the right to speak and to assert their reasons. The public reason consists of the forms of evidence and argument used in making decisions accountable to citizens by the state and to fellow citizens by other citizens. This implies the construction of “civic epistemologies” with which to evaluate procedures and decisions concerning new aspects of the application of scientific knowledge to people' lives (42).

We thus agree with Kearnes et al. (43) when they say that expert judgements don't exist in a vacuum. They arise from specific social and political contexts. To understand them, we, therefore, need to acknowledge the tacit assumptions embedded within expert knowledge claims, especially assumptions concerning how publics respond to expert advice.

In this vein, the lesson we can learn from the Covid-19 pandemic is two fold. The first idea is that the epistemic authority of experts in biomedical disciplines is fundamental and should be given priority by political authorities^31^. The second idea is that not all expert recommendations need to be automatically implemented, as some recommendations include axiological and regulatory elements that should be justified in the political process, not only epistemically but also normatively. In those cases, the decision-making process should, therefore, be civil, participatory in character, and perhaps even political, without giving up the criteria of competence and rationality.

## Author Contributions

All authors contributed equally to the writing of this manuscript.

## Conflict of Interest

The authors declare that the research was conducted in the absence of any commercial or financial relationships that could be construed as a potential conflict of interest.

## Footnotes

^1^https://www.worldometers.info/coronavirus/#countries (accessed July 1, 2020).

^2^https://www.bbc.com/news/world-asia-china-51403795 (accessed April 2020).

^3^https://www.youtube.com/watch?v=G5TZ6fTYrsE (accessed April 2020).

^4^https://www.politico.com/news/2020/03/16/coronavirus-pandemic-leadership-131540 (accessed April 2020).

^5^https://www.politico.eu/article/how-anti-vax-went-viral/ (accessed April 2020).

^6^https://www.who.int/vaccine_safety/initiative/detection/immunization_misconceptions/en/ (accessed April 2020).

^7^https://ourworldindata.org/life-expectancy (accessed April 2020).

^8^https://www.theguardian.com/politics/2015/nov/11/leaving-eu-would-be-a-disaster-british-universities-warn (accessed April 2020).

^9^https://www.project-syndicate.org/commentary/brexit-voters-ignoring-experts-by-jean-pisani-ferry-2016-07?barrier=accesspaylog (accessed April 2020).

^10^There is no value judgment in our analysis. We just want to show the areas of frequent contrast between certain political forces and the proposals made by experts and scientists.

^11^https://www.thehastingscenter.org/coronavirus-and-the-crisis-of-trust/ (accessed April 2020).

^12^Cf. https://www.vox.com/2020/2/10/21124881/coronavirus-outbreak-china-li-wenliang-world-health-organization (accessed April 2020).

^13^This was also thanks to the interventions of the World Health Organization, of internationally renowned universities and of websites such as Johns Hopkins, Centers for Disease Control and Prevention.

^14^Cf. https://www.theguardian.com/world/2020/mar/03/uk-government-coronavirus-plans-strip-fire-and-police-to-essentials-Covid-19. As of March 13, 2020, Great Britain was the only one among the main Western countries not to have closed schools and suspended sporting events, and it was also the only one not to have taken any measures involving restriction of travels, restriction of mass gatherings and domestic lockdown, https://twitter.com/lewis_goodall/status/1238242156365721609/photo/1. (accessed April 2020).

^15^https://www.bbc.com/news/uk-51865915 (accessed April 2020).

^16^CF https://www.bbc.com/news/uk-51857856 (accessed April 2020).

^17^https://www.theguardian.com/commentisfree/2020/mar/18/coronavirus-uk-expert-advice-wrong (accessed April 2020).

^18^https://it.reuters.com/article/healthcareSector/idUKL4N2BV3CA (accessed April 2020).

^19^https://www.reuters.com/article/us-health-coronavirus-britain-path-speci/special-report-johnson-listened-to-his-scientists-about-coronavirus-but-they-were-slow-to-sound-the-alarm-idUSKBN21P1VF (accessed April 2020).

^20^https://fortune.com/2020/03/14/coronavirus-uk-cases-herd-immunity-Covid-19/ (accessed April 2020).

^21^http://www.siaarti.it/SiteAssets/News/COVID19%20-%20documenti%20SIAARTI/SIAARTI%20-%20Covid-19%20-%20Clinical%20Ethics%20Reccomendations.pdf (accessed April 2020).

^22^Ibidem.

^23^https://www.nytimes.com/2020/03/31/us/coronavirus-covid-triage-rationing-ventilators.html (accessed April 2020).

^24^https://int.nyt.com/data/documenthelper/6846-alabama-triage-guidelines/02cb4c58460e57ea9f05/optimized/full.pdf#page=1 (accessed April 2020).

^25^https://dredf.org/the-illegality-of-medical-rationing-on-the-basis-of-disability/ (accessed April 2020).

^26^https://www.propublica.org/article/people-with-intellectual-disabilities-may-be-denied-lifesaving-care-under-these-plans-as-coronavirus-spreads (accessed April 2020).

^27^https://www.aapd.com/wp-content/uploads/2020/03/Covid-19-Response-Package.pdf (accessed April 2020); https://www.disabilityscoop.com/2020/03/30/trump-administration-says-disability-no-reason-to-deny-Covid-19-care/28065/ (accessed April 2020).

^28^https://gizmodo.com/alabama-disavows-plan-to-deny-ventilators-to-Covid-19-p-1842770059 (accessed April 2020).

^29^https://www.theverge.com/2020/4/10/21216484/google-apple-coronavirus-contract-tracing-bluetooth-location-tracking-data-app (accessed April 2020).

^30^https://techcrunch.com/2020/04/10/apple-and-google-are-launching-a-joint-covid-19-tracing-tool/ (accessed April 2020).

^31^https://theconversation.com/we-should-listen-to-coronavirus-experts-but-local-wisdom-counts-too-134034 (accessed April 2020).
